# Associations between different tau-PET patterns and longitudinal atrophy in the Alzheimer’s disease continuum: biological and methodological perspectives from disease heterogeneity

**DOI:** 10.1186/s13195-023-01173-1

**Published:** 2023-02-22

**Authors:** Rosaleena Mohanty, Daniel Ferreira, Agneta Nordberg, Eric Westman, for the Alzheimer’s Disease Neuroimaging Initiative

**Affiliations:** 1 Division of Clinical Geriatrics, Center for Alzheimer Research. Department of Neurobiology, Care Sciences and Society, Karolinska Institutet, Blickagången 16, 14152 Huddinge, Sweden; 2 Department of Radiology, Mayo Clinic, Rochester, MN USA; 3 Theme Aging, Karolinska University Hospital, Stockholm, Sweden; 4 Department of Neuroimaging, Centre for Neuroimaging Sciences, Institute of Psychiatry, Psychology and Neuroscience, King’s College London, London, UK

**Keywords:** Alzheimer’s disease continuum, Heterogeneity, Tau-PET, Subtypes, Patterns, Longitudinal MRI

## Abstract

**Background:**

Subtypes and patterns are defined using tau-PET (tau pathology) and structural MRI (atrophy) in Alzheimer’s disease (AD). However, the relationship between tau pathology and atrophy across these subtypes/patterns remains unclear. Therefore, we investigated the biological association between baseline tau-PET patterns and longitudinal atrophy in the AD continuum; and the methodological characterization of heterogeneity as a continuous phenomenon over the conventional discrete subgrouping.

**Methods:**

In 366 individuals (amyloid-beta-positive cognitively normal, prodromal AD, AD dementia; amyloid-beta-negative cognitively normal), we examined the association between tau-PET patterns and longitudinal MRI. We modeled tau-PET patterns as a (a) continuous phenomenon with key dimensions: *typicality* and *severity*; and (b) discrete phenomenon by categorization into patterns: *typical*, *limbic predominant*, *cortical predominant* and *minimal tau*. Tau-PET patterns and associated longitudinal atrophy were contextualized within the Amyloid/Tau/Neurodegeneration (A/T/N) biomarker scheme.

**Results:**

Localization and longitudinal atrophy change vary differentially across different tau-PET patterns in the AD continuum. Atrophy, a downstream event, did *not always* follow a topography akin to the corresponding tau-PET pattern. Further, heterogeneity as a continuous phenomenon offered an alternative and useful characterization, sharing correspondence with the conventional subgrouping. Tau-PET patterns also show differential A/T/N profiles.

**Conclusions:**

The site and rate of atrophy are different across the tau-PET patterns. Heterogeneity should be treated as a continuous, not discrete, phenomenon for greater sensitivity. Pattern-specific A/T/N profiles highlight differential multimodal interactions underlying heterogeneity. Therefore, tracking multimodal interactions among biomarkers longitudinally, modeling disease heterogeneity as a continuous phenomenon, and examining heterogeneity across the AD continuum could offer avenues for precision medicine.

**Supplementary Information:**

The online version contains supplementary material available at 10.1186/s13195-023-01173-1.

## Introduction

The biological framework of Alzheimer’s disease (AD) recognizes beta-amyloid (Aβ), tau, and neurodegeneration as the characteristic biomarkers in disease pathogenesis [1]. Among the AD hallmarks, spread of Aβ in the brain is rather diffuse whereas the accumulation of tau occurs in a more ordered manner [2]. Occurrence of neurodegeneration downstream to Aβ and tau has impelled several investigations on the relationship among these biomarkers, suggesting a closer association between neurodegeneration and tau than neurodegeneration and Aβ [3–6].

Biological heterogeneity in AD manifests as distinct patterns of biomarkers in the cognitively normal and prodromal stages. In contrast to the biomarker-based *subtypes* which are typically found at the dementia stage and are unlikely to change, biomarker-based *patterns* are more likely to evolve and change over time as the disease progresses. Neuroimaging studies have shown topographical conformity and association between tau pathology from tau positron emission tomography (tau-PET) and longitudinal brain atrophy-based neurodegeneration from magnetic resonance imaging (MRI), in cognitively unimpaired individuals [7], prodromal AD and/or AD dementia [4, 8, 9], and clinical subtypes of AD [10, 11]. A critical caveat, however, is the failure to account for heterogeneity in tau-PET topography at a given disease stage (i.e., tau patterns in cognitively normal and prodromal stages or subtypes at dementia stage) [12–16]. The relationship between tau-PET patterns and atrophy remains unexplored and is critical for precision medicine.

To this end, our study aims to provide two complementary perspectives on this issue (see Supplementary Figure 1 for study design): (a) *Biological perspective*: we investigated the association between different tau-PET patterns and longitudinal atrophy in the AD continuum (cognitively normal, prodromal AD, AD dementia cases with Aβ pathology); and (b) *Methodological perspective*: we characterized tau-PET patterns on a continuous scale inspired by the recent conceptual framework [17], compared to and extending beyond the conventional characterization of discrete categorization [14–16, 18]. This continuous-scale operationalization comprises two key dimensions including typicality (spanning from limbic predominant to cortical predominant patterns) and severity (spanning from typical AD to minimal tau patterns). Together, these dimensions represent the heterogeneity of an individual as a combination of protective factors, risk factors, and concomitant comorbid pathologies in AD.

Corresponding to these two perspectives, we hypothesized that (a) *biologically*, tau-PET patterns would modulate the association between baseline tau-PET and longitudinal atrophy differentially; and (b) *methodologically*, treating heterogeneity (i.e., the different tau-PET patterns) on a continuous scale over a discrete scale can potentially be more efficient for future research.

## Materials and methods

### Participants

Participants were chosen from the Alzheimer’s disease neuroimaging initiative (ADNI; launched in 2003; PI: Michael W. Weiner; http://adni.loni.usc.edu/), aimed at measuring the progression of prodromal, early AD using biomarkers and clinical and neuropsychological assessments. We included 366 individuals including 173 Aβ+ individuals (98 cognitively normal, 50 prodromal AD including both early and late mild cognitive impairment, and 25 AD dementia) and 193 Aβ− cognitively normal individuals. Aβ status was determined through amyloid PET (florbetapir standardized uptake value ratio or SUVR cutpoint = 1.11 [19]; or florbetaben SUVR cutpoint = 1.08 from http://adni.loni.usc.edu/). The detailed inclusion and exclusion criteria for the ADNI can be found at http://adni.loni.usc.edu/methods/. All individuals had tau-PET and MRI cross-sectionally (*baseline*). The interval between tau-PET and MRI at baseline was about 90 days (except in 5 prodromal AD and 3 AD dementia patients, >90 days). While longitudinal tau-PET were not assessed owing to the limited availability, longitudinal MRI were included both *retrospectively* (*N*=167) and *prospectively* (*N*=178). All procedures performed in the ADNI involving human participants were in accordance with the ethical standards of the local institutional review boards and with the 1964 Helsinki declaration and its later amendments. Informed consent was obtained from all individual participants included in the study.

### Neuroimaging

#### Tau-PET

Tau-PET were collected on PET/CT scanners. [^18^F] AV-1451 was injected with a dosage of 370 MBq (10.0 mCi) ± 10% and scans were acquired between 75 and 105 min post-injection. Dynamic acquisition was 30 min long with 6×5 min frames. Tau-PET scans were processed using the PetSurfer Toolbox [20] within FreeSurfer 6.0.0. AV-1451 images were co-registered onto the cross-sectionally processed MRI and visually assessed for alignment. We chose to perform and report only partial volume corrected (PVC) values using the symmetric geometric matrix method [21] based on a previous subtyping study which demonstrated reasonable agreement between PVC and non-PVC data in this cohort [15]. Regional AV-1451 signal was quantified in terms of the SUVR, computed with the cerebellum gray matter as the reference in the same 68 brain areas as MRI and represented tau pathology in the brain.

#### MRI

MRI, collected on 3.0 T scanners, were 3-D accelerated T1-weighted sequences acquired sagittally with voxel size 1.1×1.1×1.2 mm^3^. The MRI data were processed through TheHiveDB system [22] using FreeSurfer 6.0.0 (http://freesurfer.net/). Data were first preprocessed through the cross-sectional FreeSurfer stream. Resulting segmentations were visually screened for quality control. Screened scans were included for further preprocessing through the longitudinal FreeSurfer stream [23]. Automatic region of interest parcellation yielded thickness in 68 cortical structures [24] representing brain atrophy.

### Regions of interest

We examined tau-PET SUVR and thickness, averaged bilaterally, in two key regions of interest (Supplementary Figure 2). These regions are relevant to the understanding of biological heterogeneity in tau pathology as described in the seminal work by Murray et al. [12], and have been consistently identified across several tau-PET/MRI-based subtyping studies in AD [14, 15, 25, 26]. Specifically, the medial temporal lobe was represented by the *entorhinal cortex* unless specified otherwise. Hippocampus was excluded as it may suffer from off-target binding with the current tau-PET tracer [27, 28]. *Neocortex* included the middle frontal, inferior parietal, and superior frontal regions unless specified otherwise. Global tau-PET SUVR was calculated by averaging across all brain regions (except hippocampus).

### Characterization of tau-PET patterns

In line with the *methodological* aim of this study, we investigated *different tau-PET patterns* in the AD continuum with two different characterizations of baseline tau-PET:

#### Methodological perspective I: Tau-PET patterns on a continuous scale

We quantified two dimensions of tau-PET patterns in our cohort, measured on a continuous scale: *typicality*, proxied by the ratio of entorhinal tau-PET SUVR to neocortical tau-PET SUVR (hereon referred to as *E:N*) to capture the atypical patterns, similar to the index in the original neuropathological study [12]; and *severity*, proxied by the global tau-PET SUVR to capture the overall disease burden or stage. Our choice of using E:N to represent typicality was motivated by the following: the ratio of neurofibrillary tangles observed at autopsy in the medial temporal lobe relative to those in the neocortex captures atypical patterns [12]; subsequent in vivo studies using tau-PET (AV-1451) have shown the promise of this measure in quantifying heterogeneity [14, 18]; the recently proposed conceptual framework for AD subtypes based on a meta-analysis of various tau pathology- and atrophy-based studies support such a measure to investigate disease heterogeneity [17]; and the distribution of entorhinal and neocortical tau-PET SUVR in the AD continuum which reflects both individuals with relatively greater tau-PET SUVR in the entorhinal than in the neocortex and vice versa (Supplementary Figure 3). Global tau-PET SUVR was used to represent severity as it is well-known to correlate with the different disease stages and cognitive decline in AD [29].

#### Methodological perspective II: Tau-PET patterns on a discrete scale

We translated a MRI-based subtyping method [26] to tau-PET, using the healthy (Aβ−) reference group to characterize patterns within our target population as this method does not rely on assumptions on the within-population distribution (Supplementary Section 1). Briefly, this method compares the deviation of the entorhinal and neocortical SUVR of each individual relative to the healthy group and classifies the individual into one of four discrete patterns: *typical AD*, *limbic predominant*, *cortical predominant*, or *minimal tau*. We have previously identified tau-PET subtypes in AD dementia using this approach and reported how this method relates to other discrete-scale operationalizations [15]. Since a cortical predominant pattern can be heterogeneous in itself [16, 30, 31], we further investigated the contribution of different cortical regions (frontal, parietal, temporal, and occipital) to this pattern [30].

### Statistical analysis

We compared the clinical groups within the AD continuum and the healthy individuals by demographics and clinical variables [32, 33] using hypothesis testing (Kruskal–Wallis test for the continuous variables; Fisher exact test for the nominal variables).

In line with the *biological* aim of this study, we investigated the association between tau-PET SUVR and longitudinal atrophy for the tau-PET patterns as follows:

#### Biological perspective I: Association between baseline tau-PET patterns (continuous scale) and longitudinal atrophy

Testing our hypothesis that tau-PET patterns on the continuous scale may be differentially associated with longitudinal atrophy, we modeled tau-PET patterns (typicality and severity) and estimated regional thickness changes using linear mixed effects model. We used individual-specific intercepts and the fixed effects included time (centered at baseline, T_B_), age, typicality, severity, interaction of each of typicality and severity with time. The dependent variable was longitudinal regional thickness. For visualization, we assessed atrophy changes stratified by typicality (limbic predominant versus cortical predominant tau-PET patterns) and by severity (typical AD versus minimal tau patterns). Per stratification, we computed the thickness change over time across individuals across retrospective, baseline, and prospective timepoints.

#### Biological perspective II: Association between baseline tau-PET patterns (discrete scale) and longitudinal atrophy

Testing our hypothesis that tau-PET patterns on the discrete scale may be differentially associated with longitudinal atrophy, we modeled tau-PET patterns (typical AD, limbic predominant, cortical predominant, or minimal tau) and estimated regional thickness changes using linear mixed effects model. We used individual-specific intercepts and the fixed effects included time (centered at baseline, T_B_), age, tau-PET pattern (centered at minimal tau pattern), and interaction of each of the tau-PET patterns with time. The dependent variable was longitudinal regional thickness. For visualization, we assessed atrophy changes stratified by tau-PET patterns. Per stratification, we computed the thickness change over time across individuals for retrospective, baseline, and prospective timepoints. Topographical overlap between baseline tau-PET patterns (binarized such that higher tau-PET SUVR in the pattern than the healthy group was assigned 1) and atrophy (binarized such that *Z*-score of thickness < 0.5 was assigned 1) at each timepoint was assessed using Sørensen–Dice coefficient with values closer to 1 corresponding to greater overlap between tau-PET and atrophy patterns.

#### Biological perspectives I–II: Continuous versus discrete scale tau-PET patterns

Biological perspectives I and II were quantitatively compared to each other in two ways. First, we stratified the continuous-scale measures of typicality and severity by the discrete-scale tau-PET patterns. Second, we compared the two perspectives with a simulated likelihood ratio test between the two linear mixed effects models with 1000 replications. The continuous-scale and the discrete-scale models were compared in terms of the Akaike information criterion (AIC), Bayesian information criterion (BIC) and maximized log likelihood.

#### Biological perspective III: A/T/longitudinal-N classification of baseline tau-PET patterns

For a deeper understanding of tau-PET patterns, we analyzed the A/T/longitudinal-N (Aβ/Tau/longitudinal-atrophy-based neurodegeneration) biomarker scheme [1] across them. We dichotomized each biomarker as follows: Aβ positivity with global amyloid PET SUVR (florbetapir SUVR cutpoint = 1.11 [19]; or florbetaben SUVR cutpoint = 1.08 from http://adni.loni.usc.edu/; denoted by A+), tau positivity with regional tau-PET-based SUVR in the medial temporal and cortical regions (based on previously established cutpoints [26, 34] described in Supplementary Section 2; denoted by T+) and neurodegeneration positivity at retrospective, baseline and prospective timepoints with regional MRI-based thickness/atrophy in the medial temporal and cortical regions (based on previously established cutpoints [26, 35] described in Supplementary Section 3; denoted by N_R_+, N_B_+, N_P_+). In the main report, we include tau and neurodegeneration positivity assessed in the entorhinal cortex and the neocortex (Supplementary Figure 2). We compared the proportion (%) of A/T/longitudinal-N positivity across tau-PET patterns.

Analyses were performed using MATLAB R2020b (The MathWorks, Inc., Natick, Massachusetts, USA). Brain visualizations were generated through R v4.0.3 with ggseg (https://lcbc-uio.github.io/ggseg/).

## Results

### Participants

The study cohort comprised 366 individuals including 173 Aβ+ AD continuum and 193 Aβ− cognitively normal cases. Clinical groups in this cohort were significantly different in age, education, *APOE* ε4 carriers and global cognition (Table 1). For longitudinal MRI, the retrospective-to-baseline time interval was 2.2 ± 1 years while the baseline-to-prospective time interval was 1.3 ± 0.4 years.

**Table 1:** Characteristics of the study cohort

Diagnosis (baseline)	Healthy (Aβ−)	Cognitively normal (Aβ+)	Prodromal AD (Aβ+)	AD dementia (Aβ+)
***N*** **(baseline tau-PET, MRI)**	193	98	50	25
***N*** **(retrospective MRI)**	73	46	31	17
***N*** **(prospective MRI)**	66	57	42	13
**Age at baseline (years)**	71.9 ± 6.4 [56, 95]	75.5 ± 7.1 [62, 92]^a^	75.3 ± 7.7 [59, 92]^a^	78.2 ± 8.2 [56, 91]^a^
**Sex (% female)**	59.6	56.1	52	48
**Education (years)**	17 ± 2.3 [11, 20]^b^	16.7 ± 2.3 [12, 20]^b^	15.6 ± 2.6 [12, 20]	15.9 ± 2.6 [12, 20]
***APOE*** **ε4 carriers (%)**^**e**^	23.6	56.7^a^	62^a^	56^a^
**MMSE at baseline**^**f**^	29.3 ± 1.0 [23, 30]^c^	28.8 ± 1.5 [22, 30]^c^	27.6 ± 2.3 [19, 30]^d^	22 ± 4.2 [9, 30]^d^

Data are reported as mean ± standard deviation [minimum, maximum]. Hypothesis testing was performed using the Kruskal–Wallis test for the continuous variables and Fisher exact test for the nominal variables. ^a^ significantly different from Healthy (Aβ−); ^b^ significantly different from Prodromal AD; ^c^ significantly different from Prodromal AD and AD Dementia; ^d^ significantly different from all other groups; ^e^ Missing values = 3; ^f^ Missing values = 2; *Aβ* β-amyloid; *AD* Alzheimer’s disease; *APOE* apolipoprotein; *MMSE* Mini-Mental State Examination

### Characterization of tau-PET patterns

#### Methodological perspective I: Tau-PET patterns on a continuous scale

**Fig. 1:**
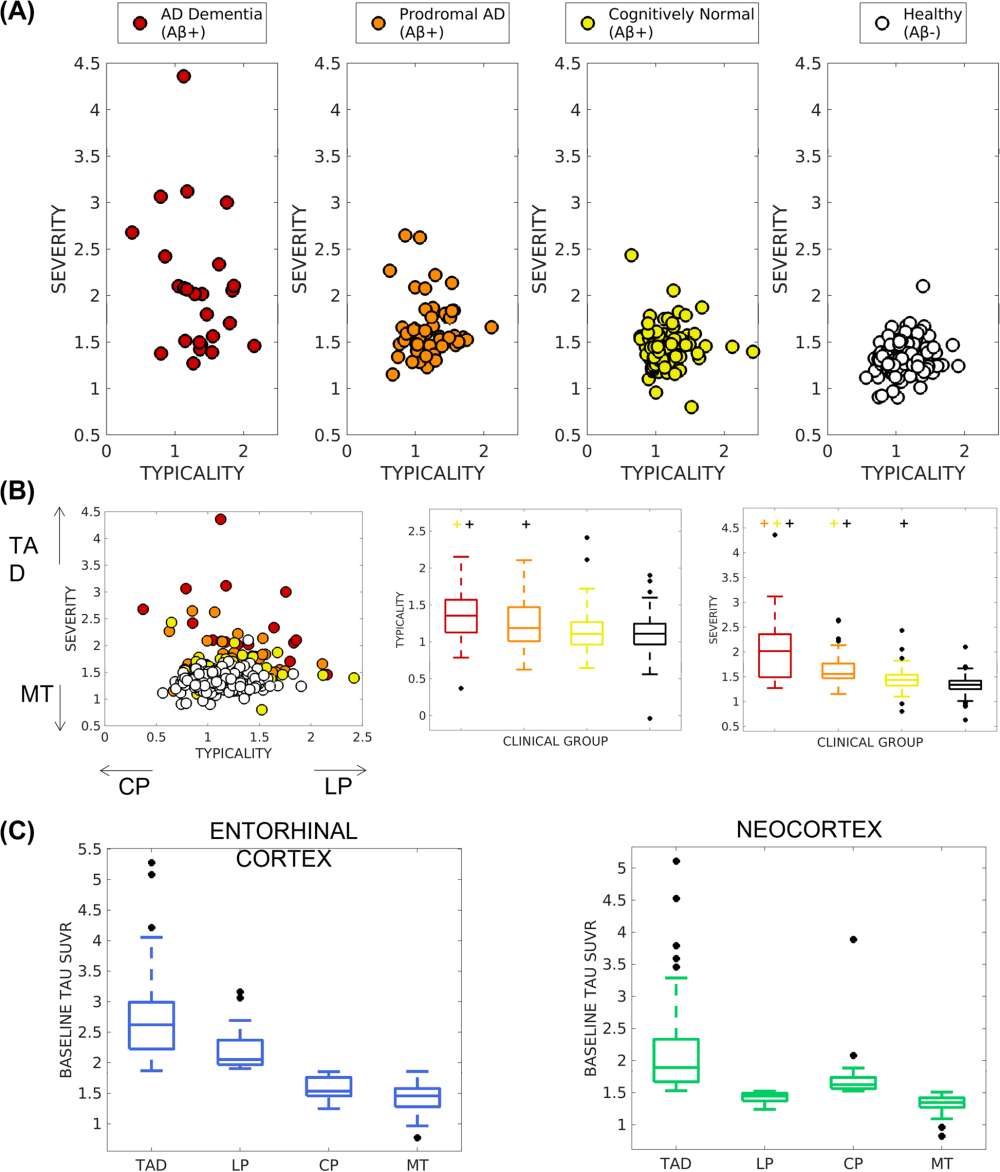
Baseline tau-PET patterns in the AD continuum characterized on a **A, B** continuous scale and **C** discrete scale. **A** Tau-PET patterns were assessed on a continuous scale across the AD continuum (including cognitively normal, prodromal AD, and AD dementia), shown separately in each clinical group for visual comparison. **B** Tau-PET patterns assessed on a continuous scale, shown relative to the other clinical groups (left) with the relative tendency of typicality (in terms of CP or LP) and severity (in terms of TAD or MT) shown for correspondence with **C**; variability of typicality (center) and severity (right) across the clinical groups is presented; significant difference (*p* < 0.05) for each group compared to the other groups is indicated by “+” above each boxplot. **C** Tau-PET patterns, assessed on a discrete scale in the AD continuum relative to the healthy group, identified four patterns whose tau-PET SUVR are shown in the entorhinal cortex (left) and the neocortex (right); by definition, there are significant differences (*p* < 0.05) between all pairs of patterns. AD = Alzheimer’s disease; TAD = typical AD pattern; LP = limbic predominant pattern; CP = cortical predominant pattern; MT = minimal tau

We defined tau-PET patterns by *typicality* (ratio of entorhinal tau-PET SUVR to neocortical tau-PET SUVR denoted by *E:N*) and *severity* (global tau-PET SUVR). Figure 1A, B shows the distribution of tau-PET patterns in terms of typicality and severity on a continuous scale. Within the AD continuum, both dimensions showed the lowest variance in the Aβ+ cognitively normal (*σ*^2^=0.07 for typicality, *σ*^2^=0.05 for severity), an intermediate variance in prodromal AD (*σ*^2^=0.09 for typicality, *σ*^2^=0.1 for severity) and the highest variance in AD dementia (*σ*^2^=0.16 for typicality, *σ*^2^=0.52 for severity) (Fig. 1B). Overall, there was no significant association between typicality and severity in tau-PET (*r*=0.003, *p*=0.97).

#### Methodological perspective II: Tau-PET patterns on a discrete scale

We adapted a MRI-based subtyping method [26] to tau-PET as demonstrated previously [15] to characterize four distinct patterns based on the entorhinal and neocortical tau-PET SUVR (Supplementary Section 1). Figure 1C presents these four discrete tau-PET patterns: 33% typical AD pattern (*N*=57), 12% limbic predominant pattern (*N*=21), 18% cortical predominant pattern (*N*=31), and 37% minimal tau pattern (*N*=64). Demographic and clinical characteristics across patterns are summarized in Table 2. The tau-PET patterns were significantly different in education, global, and composite cognitive scores. Longitudinal decline in domain-specific composite scores were larger than those in global cognition. We observed significant and differential associations between longitudinal changes in global/composite cognitive scores and baseline tau as well as longitudinal atrophy change (Supplementary Section 4, Supplementary Table 1). For the cortical predominant pattern, a further analysis of the involvement of different cortical regions (frontal, parietal, temporal, occipital) showed that this pattern was primarily characterized by lower medial temporal tau-PET SUVR compared to the other patterns (*p* < 0.0021) while being comparable to healthy individuals (Supplementary Figure 4).

**Table 2:** Characteristics of the tau-PET patterns (discrete scale)

Subtypes at baseline (***N***=173)	TAD (***N***=57)	LP (***N***=21)	CP (***N***=31)	MT (***N***=64)
**AD dementia/ prodromal AD/ cognitively normal (%)**	28/42/30	24/24/52	6/32/61	2/17/80
**Typicality (mean (SD))**	1.3 (0.3) ^a, b, c^	1.6 (0.3) ^a, b^	0.9 (0.2) ^a^	1.1 (0.2)
**Severity (mean (SD))**	1.9 (0.5) ^a, b, c^	1.5 (0.1) ^a, b^	1.6 (0.2) ^a^	1.3 (0.1)
**Age at baseline (years)**	76.3 ± 7.3 [62, 90]	76.3 ± 6.4 [65, 90]	76.6 ± 7.8 [56, 91]	74.9 ± 7.8 [59, 92]
**Sex (% female)**	64.9	42.9	54.8	46.9
**Education (years)**	15.9 ± 2.3 [12, 20] ^a^	15.6 ± 2.6 [12, 20] ^a^	16.1 ± 2.4 [12, 20]	17 ± 2.4 [12, 20]
***APOE*** **ε4 carriers (%)**	66.7	57.1	58.1	50.8
**Retrospective MMSE**	27.10 ± 3.15 [17, 30] ^a^	27.25 ± 1.75 [24, 29] ^a^	27.74 ± 2.40 [23, 30] ^a^	29 ± 1.25 [26, 30]
**Baseline MMSE**	26.1 ± 4.3 [9, 30] ^a^	26.8 ± 3.1 [20, 30] ^a, b^	28.2 ± 2.1 [21, 30]	28.6 ± 1.9 [17, 30]
**Prospective MMSE**	25.6 ± 3.90 [13, 30] ^a, b^	26.5 ± 4.22 [16, 30] ^a^	28.37 ± 2.09 [21, 30]	28.81 ± 2.26 [17, 30]
**Retrospective ADNI-MEM**	0.33 ± 1.16 [−2.31, 2.08] ^a^	0.31 ± 0.61 [−0.55, 1.51] ^a^	0.73 ± 0.7 [−0.56, 1.95] ^a^	1.19 ± 0.66 [−0.04, 2.57]
**Baseline ADNI-MEM**	0.10 ± 0.96 [−2.38, 2.01] ^a, b^	0.19 ± 0.62 [−1.19, 1.21] ^a^	0.55 ± 0.66 [−0.84, 1.69] ^a^	0.93 ± 0.56 [−0.40, 2.10]
**Prospective ADNI-MEM**	−0.03 ± 1.08 [−2.56, 2.08] ^a, b^	0.10 ± 0.82 [−1.39, 1.45] ^a^	0.53 ± 0.69 [−0.88, 1.79] ^a^	1.02 ± 0.70 [−0.24, 2.51]
**Retrospective ADNI-EF**	0.51 ± 0.91 [−1.37, 2.25] ^a^	0.28 ± 1.19 [−1.48, 2.12] ^a^	0.78 ± 0.72 [−0.68, 2.47]	1.07 ± 0.79 [−0.56, 2.73]
**Baseline ADNI-EF**	0.04 ± 1.12 [−2.43, 2.99] ^a, c^	0.62 ± 1.01 [−1.40, 2.00]	0.43 ± 0.88 [−2.78, 2.23] ^a^	0.94 ± 0.87 [−2.31, 2.72]
**Prospective ADNI-EF**	0.0016 ± 1.19 [−2.91, 2.23] ^a^	0.61 ± 0.83 [−0.83, 1.99]	0.24 ± 0.95 [−2.39, 1.59] ^a^	0.81 ± 0.92 [−2.25, 2.99]

Data are reported as mean ± standard deviation [minimum, maximum]. Hypothesis testing was performed using the Kruskal–Wallis test for the continuous variables and Fisher exact test for the nominal variables. ^a^ significantly different from minimal tau pattern; ^b^ significantly different from cortical predominant pattern; ^c^ significantly different from limbic predominant pattern; *AD* Alzheimer’s disease; *SD* standard deviation; *TAD* typical AD pattern; *LP* limbic predominant pattern; *CP* cortical predominant pattern; *MT* minimal tau pattern; *APOE* apolipoprotein; *MMSE* Mini-Mental State Examination; *ADNI-MEM* composite cognitive scores for memory; *ADNI-EF* composite cognitive scores for executive function

### Association between baseline tau-PET patterns and longitudinal atrophy

#### Biological perspective I: Tau-PET patterns on a continuous scale

**Fig. 2:**
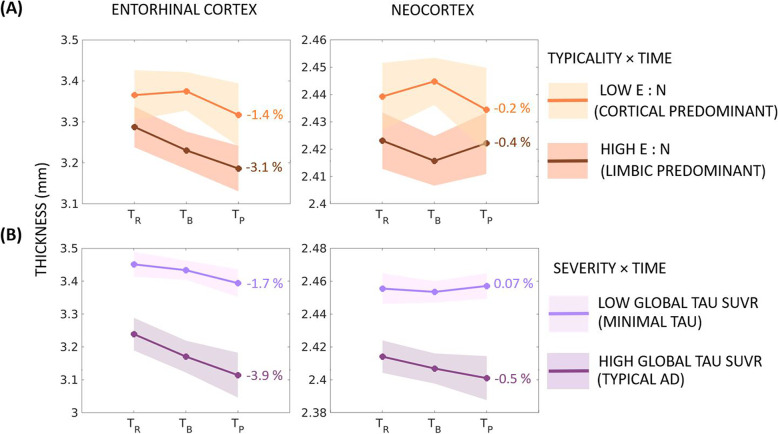
Longitudinal changes in atrophy relative to baseline tau-PET patterns (continuous scale) in the AD continuum. Estimated longitudinal atrophy (thickness) estimated by linear mixed effects model for the entorhinal cortex and neocortex: **A** stratified by levels of typicality (the low/high groups were computed by median split in typicality); **B** stratified by levels of severity (the low/high groups were computed by median split in severity). Shaded regions represent the 95% confidence interval; percentages indicate the overall change in thickness per group over the period between retrospective and prospective timepoints; E:N = ratio of average entorhinal tau-PET SUVR to average neocortical tau-PET SUVR; SUVR = standardized uptake value ratio

Figure 2 and Table 3 show the estimated changes in longitudinal regional thickness using baseline tau-PET patterns. Assessing our primary hypothesis of differential association of tau-PET patterns (typicality and severity) with longitudinal atrophy, we assessed the interaction of each of typicality and severity with time on estimation of regional thickness change (%) as described below.

**Table 3:** Estimated rate of atrophy change as a function of baseline tau-PET patterns (continuous scale)

Fixed effects	Entorhinal cortex	Neocortex
	Estimate (SE)	Estimate (SE)
Intercept	5.24 (0.25) **	2.86 (0.08) **
Age	−0.01 (0.003) **	−0.003 (0.0009) **
T_R_	−0.28 (0.04) **	−0.05 (0.01) *
T_P_	0.23 (0.04) **	0.04 (0.01) *
Typicality	−0.26 (0.08) **	−0.04 (0.02)
Severity	−0.45 (0.06) **	−0.08 (0.02) **
Typicality_B_ × T_R_	0.14 (0.02) **	0.02 (0.01)
Typicality_B_ × T_P_	−0.1 (0.02) **	−0.0009 (0.01)
Severity_B_ × T_R_	0.1 (0.01) **	0.02 (0.01) *
Severity_B_ × T_P_	−0.1 (0.02) **	−0.03 (0.01) **

Changes in atrophy (thickness) are estimated by the linear mixed effects model with individual-specific intercepts. The linear mixed effects model was centered at T_B_. Longitudinal atrophy was modeled as the dependent variable. Age, time, Typicality_B_, Severity_B_, and interactions with time were modeled as fixed effects. The significant effects corresponding to *p*≤0.001 and *p*≤0.01 are marked by ** and * respectively. *SE* standard error in coefficient; *T*_*R*_ retrospective timepoint; *T*_*P*_ prospective timepoint, *Typicality*_*B*_ baseline typicality (proxied by E:N), *Severity*_*B*_ baseline severity (proxied by global tau-PET SUVR)

##### Typicality × Time interaction

Table 3 shows that the estimated longitudinal thickness changes over the three timepoints were significant for the entorhinal cortex but not the neocortex. Stratifying by typicality (E:N) showed faster entorhinal thinning for higher E:N (limbic predominant pattern, −3.1%/year) than for lower E:N (cortical predominant pattern, −1.4%/year) (Fig. 2A). On the other hand, neocortical thinning over time was comparable between the limbic predominant pattern (−0.4%/year) and cortical predominant pattern (−0.2%/year).

##### Severity × Time interaction

Table 3 shows that the estimated longitudinal thickness changes over the three timepoints were significant for both the entorhinal cortex and the neocortex. Stratifying by severity (global tau SUVR) showed faster entorhinal thinning for higher global tau SUVR (typical AD pattern, −3.9%/year) than for lower global tau SUVR (minimal tau pattern, −1.7%/year) (Fig. 2B). Similarly, faster neocortical thinning was observed for the typical AD (−0.5%/year) compared to the minimal tau pattern (−0.07%/year).

#### Biological perspective II: Tau-PET patterns on a discrete scale

**Fig. 3:**
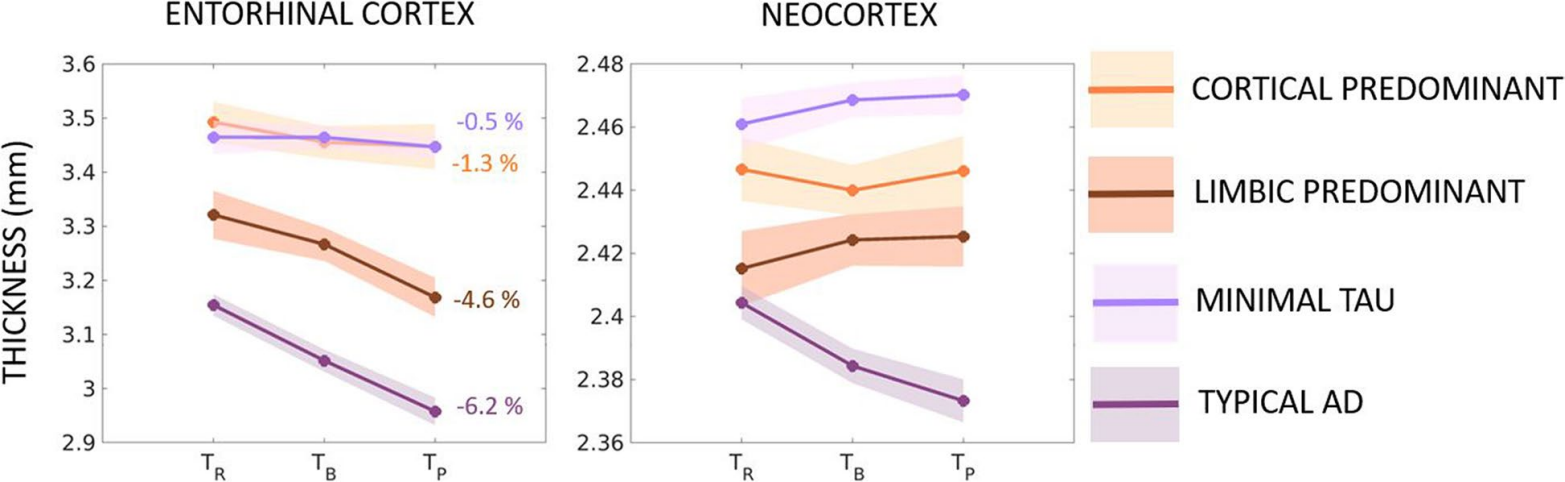
Longitudinal changes in atrophy relative to baseline tau-PET patterns (discrete scale) in the AD continuum. Estimated longitudinal atrophy (thickness) estimated by linear mixed effects model for the entorhinal cortex and neocortex stratified by levels of tau-PET patterns on the discrete scale including typical AD, limbic predominant, cortical predominant, and minimal tau patterns. Shaded regions represent the 95% confidence interval; percentages indicate the overall change in thickness per group over the period between retrospective and prospective timepoints

Figure 3 and Table 4 show the tau-PET patterns on the discrete scale and how they estimate the changes in longitudinal regional thickness. Assessing our primary hypothesis of differential association of tau-PET patterns (typical AD, limbic predominant, cortical predominant, minimal tau) with longitudinal atrophy, we assessed the interaction of each pattern with time on estimation of regional thickness change (%) relative to minimal tau pattern as the reference as described below.

**Table 4:** Estimated rate of atrophy change as a function of baseline tau-PET patterns (discrete scale)

Fixed effects	Entorhinal cortex	Neocortex
	Estimate (SE)	Estimate (SE)
Intercept	4.25 (0.23) **	2.68 (0.07) **
Age	−0.01 (0.003) **	−0.003 (0.001) *
T_R_	−0.004 (0.02)	−0.009 (0.01)
T_P_	−0.01 (0.02)	0.004 (0.01)
TAD_B_	−0.4 (0.06) **	−0.08 (0.02) **
LP_B_	−0.18 (0.08) *	−0.04 (0.02)
CP_B_	0.01 (0.07)	−0.02 (0.02)
TAD_B_ × T_R_	0.1 (0.03) **	0.03 (0.01) *
TAD_B_ × T_P_	−0.08 (0.03) *	−0.01 (0.01)
LP_B_ × T_R_	0.1 (0.04) *	0.01 (0.02)
LP_B_ × T_P_	−0.09 (0.04) *	−0.002 (0.01)
CP_B_ × T_R_	0.02 (0.03)	0.01 (0.01)
CP_B_ × T_P_	0.001 (0.03)	0.002 (0.01)

Changes in atrophy (thickness) are estimated by the linear mixed effects model with individual-specific intercepts. The linear mixed effects model was centered at T_B_ and minimal tau pattern. Longitudinal atrophy was modeled as the dependent variable. Age, time, tau-PET pattern at baseline (TAD_B_, LP_B_, CP_B_), and interactions of the patterns with time were modeled as fixed effects. The significant effects corresponding to *p*≤0.001 and *p*≤0.05 are marked by ** and * respectively. *SE* standard error in coefficient; *T*_*R*_ retrospective timepoint; *T*_*P*_ prospective timepoint, *TAD*_*B*_ typical AD pattern at baseline, *LP*_*B*_ limbic predominant pattern at baseline, *CP*_*B*_ cortical predominant pattern at baseline

##### Pattern × Time interaction

Table 4 shows that the estimated longitudinal thickness changes over the three timepoints were significant for some but not all patterns in the entorhinal and neocortex. In the entorhinal cortex, typical AD (−6.2%/year) and limbic predominant (−4.6%/year) but not the cortical predominant (−1.3%/year) pattern showed faster thinning compared to minimal tau pattern (−0.5%/year) (Fig. 3). In the neocortex, estimated longitudinal thickness changes from retrospective-to-baseline timepoints were significant for the typical AD pattern (−1.3%/year) only compared to the minimal tau pattern (0.4%/year) (Fig. 3).

**Fig. 4:**
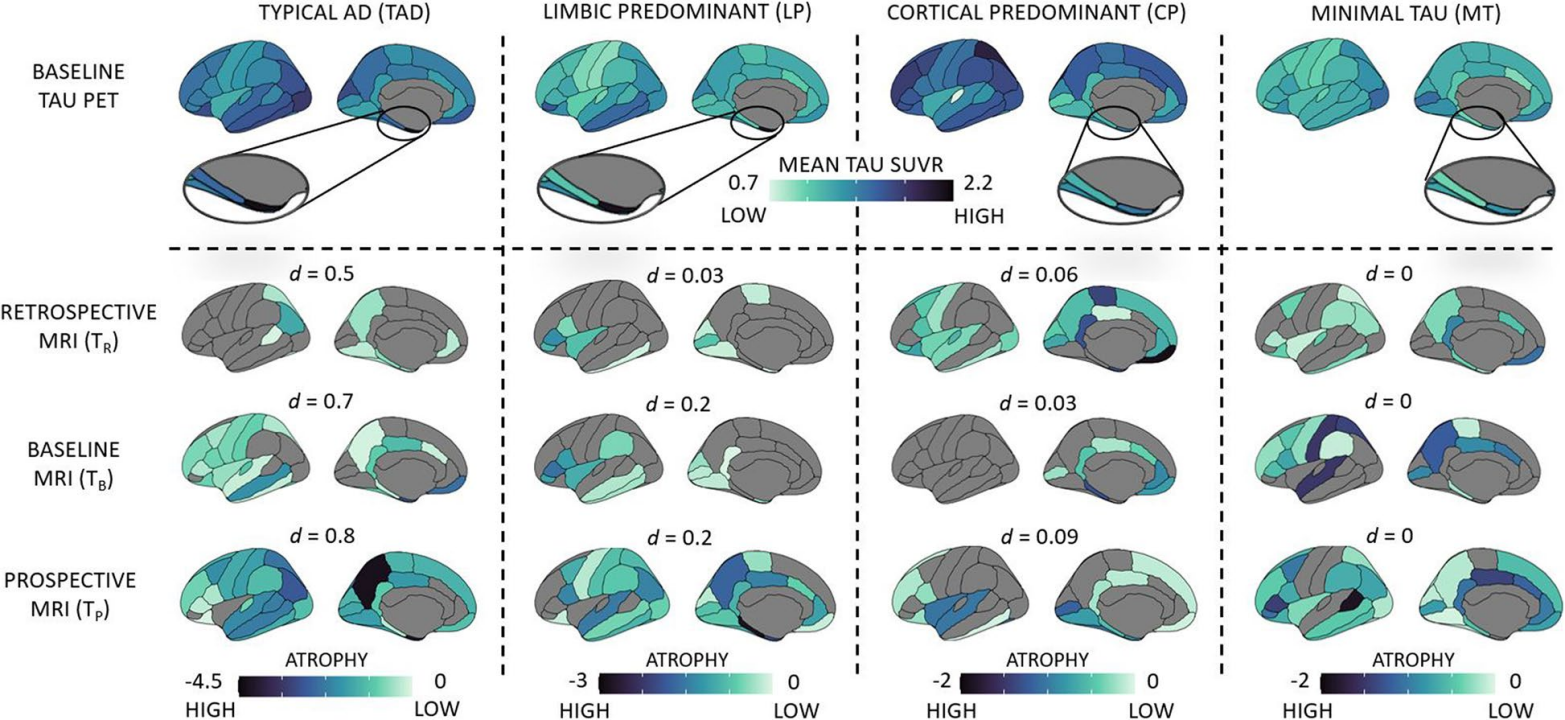
Baseline tau-PET patterns (discrete scale) and corresponding longitudinal atrophy in the AD continuum. Top panel: Topography of baseline tau-PET SUVR in four discrete tau-PET patterns in AD continuum (*N* = 173). The zoomed in view shows the tau-PET SUVR in the entorhinal cortex. Darker (dark blue) colors represent elevated tau-PET SUVR; bottom panels: topography of atrophy, measured in terms of longitudinal thickness (*Z*-score) corresponding to each tau-PET pattern, in a subcohort of AD continuum, tracked across retrospective, baseline and prospective timepoints (*N* = 61). Darker (dark blue) colors represent higher atrophy (thinner cortex). All cortical maps correspond to the left hemisphere (similar patterns were observed in the right hemisphere). *Z*-scores below 0 represent regional thinning. Sørensen–Dice coefficient comparing the topographical overlap between the tau-PET pattern at baseline and atrophy at each timepoint is reported as *d*. AD = Alzheimer’s disease; T_R_ = retrospective timepoint; T_B_ = baseline timepoint; T_P_ = prospective timepoint; TAD = typical AD; LP = limbic predominant; CP = cortical predominant; MT = minimal tau; *d* = Sørensen–Dice coefficient

Figure 4 (top panel) shows the topography (mean tau SUVR) of the discrete tau-PET patterns at baseline (*N*=173): typical AD pattern had elevated tau-PET SUVR in the medial temporal lobe and in the remaining cortex; limbic predominant pattern had elevated tau-PET SUVR in the entorhinal cortex compared to the remaining cortex; cortical predominant pattern had elevated tau-PET SUVR in the neocortex compared to the entorhinal cortex; minimal tau pattern did not show marked elevation of tau-PET SUVR in any region. Figure 4 (bottom panels) presents the longitudinal topography of regional thinning *within* each tau-PET pattern in a subcohort (*N*=61), tracked across all timepoints. Visually, regional thinning appeared more pronounced at later timepoints. Topography of tau-PET SUVR elevation shared greater similarity with the topography of regional thinning over time for typical AD and limbic predominant patterns (relatively higher values of Sørensen–Dice coefficient, *d*), but not for cortical predominant and minimal tau patterns (relatively lower values of Sørensen–Dice coefficient, *d*).

#### Biological perspectives I–II: Continuous- versus discrete-scale tau-PET patterns

Supplementary Figure 5 shows the continuous measures of typicality and severity, stratified by the discrete-scale tau-PET patterns. This plot shows that the continuous-scale perspective indicates not only the degree of atypicality of each individual along typicality, but also informs about how advanced each individual is the disease stage along severity. The discrete-scale perspective only provides the categorization of each individual into one of the four patterns with no additional information on where the individual may lie in relation to others in the same pattern. In Table 2, stratified values of both typicality and severity in the discrete-scale tau-PET patterns showed significant differences between all pairs of patterns. Particularly, the cortical predominant pattern was both more atypical (low typicality) and more severe (high severity) compared to the limbic predominant pattern, suggesting that these two may indeed represent distinct tau-PET patterns while also being at different disease stages. Further, a simulated likelihood ratio test compared the linear mixed effects models based on the continuous and discrete-scale tau-PET patterns (Tables 3 and 4). Both for the entorhinal and the neocortical regions, we noted *p*-value < 0.05, suggesting that the model with continuous-scale tau-PET patterns was significantly better than the one with discrete-scale tau-PET patterns in estimating longitudinal atrophy. This result was also supported by the lower values of AIC and BIC and higher values of maximized log likelihood for the continuous-scale tau-PET patterns (Table 5).

**Table 5:** Comparison of continuous-scale and discrete-scale models of tau-PET patterns

Model comparison criterion	Entorhinal cortex		Neocortex	
	Continuous-scale model	Discrete-scale model	Continuous-scale model	Discrete-scale model
Akaike information criterion	−244.96	−186.06	−1023.3	−1001.3
Bayesian information criterion	−197.71	−127	−976.1	−942.27
Maximized log likelihood	134.48	108.03	523.67	515.67
Likelihood ratio test statistic	52.906		16.011	
*p*-value [confidence interval]	0.000999		0.000999	

#### Biological perspective III: A/T/longitudinal-N classification of baseline tau-PET patterns

**Fig. 5:**
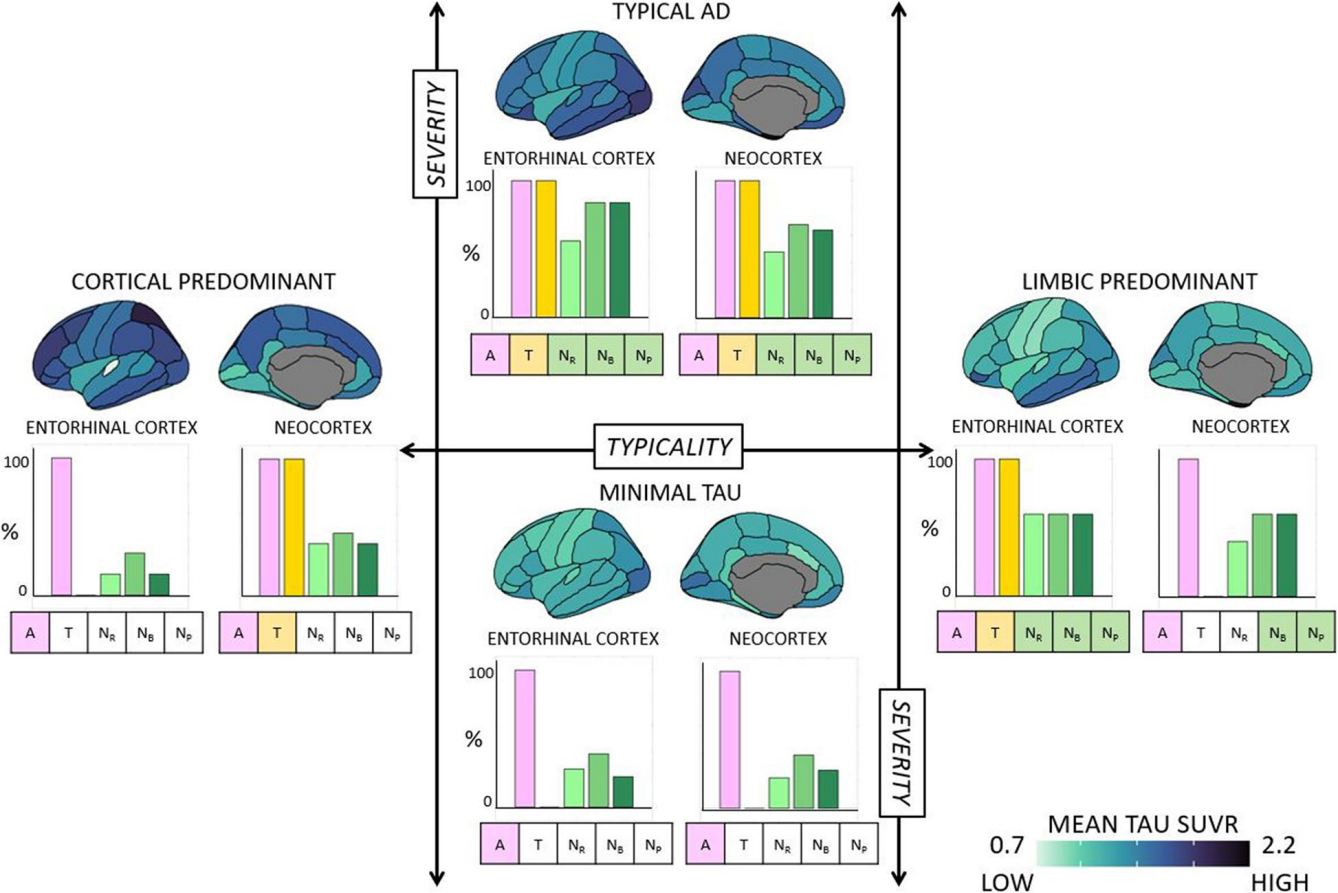
A/T/longitudinal-N classification corresponding to the tau-PET-based patterns in the AD continuum. A/T/longitudinal-N biomarker profiles for the four discrete tau-PET-based patterns were mapped in the subcohort of the AD continuum (*N*=61). Typicality (horizontal axis) and severity (vertical axis) dimensions are superposed, as proposed in the original conceptual framework [17]. A+ was determined by global Aβ-PET SUVR. T+ and longitudinal N+ were determined regionally in the entorhinal cortex and the neocortex, corresponding to the regions used to identify the tau-PET patterns [24]. For each tau-PET-based pattern, the proportion of A+, T+, and longitudinal N+ (along horizontal axis) are presented as percentages (along vertical axis) in the bar plots for the entorhinal cortex and the neocortex. Atrophy, used to represent N+, was adjusted for age at each timepoint relative to a group of healthy (Aβ−) individuals. The most prevalent A/T/longitudinal-N positive profile (≥50%) corresponding to a tau-PET-based pattern is shown in boxes under each bar plot with colored boxes. A/T/longitudinal-N = Aβ/Tau/longitudinal neurodegeneration; AD = Alzheimer’s disease; N_R_ = atrophy at retrospective timepoint; N_B_ = atrophy at baseline timepoint; N_P_ = atrophy at prospective timepoint

We examined the A/T/longitudinal-N biomarker scheme across the tau-PET patterns [26] (Supplementary Sections 2-3). Amyloid positivity, A+, was evaluated globally whereas tau positivity, T+, and longitudinal neurodegeneration positivity, [N_R_+ N_B_+ N_P_+], were evaluated regionally (Supplementary Tables 2-3). Figure 5 and Supplementary Table 4 show the most prevalent (i.e., observed in ≥50% of the individuals) A/T/longitudinal-N profile across tau-PET patterns. Tau-PET patterns showed a differential biomarker positivity profile which was region-dependent. Typical AD pattern was [A+ T+ N_R_+ N_B_+ N_P_+] in both the entorhinal cortex and neocortex; limbic predominant pattern was [A+ T+ N_R_+ N_B_+ N_P_+] in the entorhinal cortex but [A+ T− N_R_− N_B_+ N_P_+] in the neocortex; cortical predominant pattern was [A+ T− N_R_− N_B_− N_P_−] in the entorhinal cortex but [A+ T+ N_R_− N_B_− N_P_−] in the neocortex; and minimal tau was [A+ T− N_R_− N_B_− N_P_−] in both the entorhinal cortex and neocortex.

## Discussion

We investigated the association between heterogeneity in tau-PET and longitudinal neurodegeneration (atrophy) in the AD continuum. As hypothesized: (a) from a *biological perspective*, different tau-PET patterns revealed a differential association with longitudinal atrophy; and (b) from a *methodological perspective*, characterizing heterogeneity on a continuous scale may be more useful than the conventional categorization of individuals into discrete patterns. Recent studies have investigated the association between tau pathology and downstream neurodegeneration in healthy, cognitively normal, prodromal AD, and AD dementia [5, 7–9, 36–38] individuals, as well as in clinical subtypes of AD [39–41]. To our knowledge, our study is the first to characterize the role of biological heterogeneity (tau-PET patterns) as a modulator of the association between tau pathology and neurodegeneration.

The four tau-PET patterns captured by the continuous and discrete scales in our study are reminiscent of the biological tau-PET AD subtypes [14, 15, 18, 42]. With regard to the cortical predominant tau-PET pattern in particular, further analysis of different regions in the cortex revealed that this pattern had relatively lower tau burden particularly in the medial temporal regions. Compared to previous studies describing cortical predominant subtypes in tau-PET (occipital-dominant/visual variant, left hemisphere-dominant/language variant, etc.) [16, 30, 31], the cortical predominant pattern in our sample is reflective of an amnestic phenotype. In this study, we describe heterogeneity in terms of tau-PET *patterns* and not *subtypes*. *Subtypes* are conventionally reported in the advanced disease stage such as in AD dementia and may potentially be less likely to change into a different subtype. However, given that our cohort additionally included individuals at earlier disease stages such as at pre-dementia stages, there may be a possibility that the *pattern* exhibited currently may eventually evolve and transition into a *different pattern* at advanced disease stages. Thus, different tau-PET topographies which may represent current *patterns* in our cohort at early disease stage may be more appropriately described as *subtypes* in AD dementia. The value of identifying *patterns* lies in that heterogeneity in tau pathology may be detectable at early stages of the disease. Our study confirms the findings from the recent report identifying four discrete trajectories in tau-PET within AD continuum [16]. The novelty of our findings lies in the associations between baseline tau-PET and longitudinal atrophy across heterogeneity and the realization of heterogeneity as a continuous phenomenon.

The prevalence of the identified tau-PET patterns differed slightly from previous reports: on the continuous scale (Fig. 1 A, B), a large proportion of the individuals exhibited intermediate values of typicality and low values of severity (lower variance in prodromal AD and cognitively normal may suggest less heterogeneity); on the discrete scale (Fig. 4), minimal tau was the most prevalent pattern (37%) and the cortical predominant pattern (18%) was more prevalent than the limbic predominant pattern (12%). This breakdown of prevalence of the patterns is different when considering AD dementia cases alone (Table 2)—typical AD pattern was the most prevalent and minimal tau pattern was the least prevalent. Thus, the discrepancy in prevalences of tau-PET patterns is likely owing to the large proportion of individuals at early disease stages (Aβ+ cognitively normal and prodromal AD), who may have not accumulated considerable amount of tau pathology, which is typical to AD. Additionally, a current tau-PET pattern at early disease stages may likely evolve into a different pattern at a later timepoint. This may explain why the demographic/clinical profiles of our tau-PET patterns (Table 2) do not entirely conform with the expected profiles previously reported in AD [17]. Similar results have been found when characterizing heterogeneity in tau-PET in the AD continuum [16], atrophy in prodromal AD [43] and glucose-hypometabolism in prodromal AD [44]. These differing prevalences may be a function of the predominant disease stage in the cohort in addition to the cutpoints used to determine abnormality in the brain regions. Altogether, heterogeneity at preclinical and prodromal stages of AD may be similar to, albeit less pronounced than, heterogeneity in AD dementia. Modeling heterogeneity on a continuous spectrum may offer an avenue to circumvent the lack of generalizability of specific prevalences of subtypes in a disease population.

Our main finding was that tau-PET patterns showed differential association with longitudinal atrophy. On the continuous scale (Table 3), typicality was significantly associated with longitudinal atrophy in the entorhinal cortex but not the neocortex. This result highlights that tau pathology in the entorhinal cortex (signature of a limbic predominant pattern) can be tracked by longitudinal atrophy in the region, with greater atrophy seen in the highest extreme of typicality (limbic predominant pattern) [45] compared to the lowest extreme (cortical predominant pattern). However, tau pathology in the neocortex (signature of a cortical predominant pattern) cannot necessarily be tracked by longitudinal atrophy in the region, with comparable atrophy seen in the limbic predominant and cortical predominant patterns. On the other hand, severity was significantly associated with longitudinal atrophy in both the entorhinal cortex and the neocortex. This result highlights that greater tau burden in the entorhinal cortex and neocortex (signature of typical AD pattern) can be tracked with greater atrophy in these regions in the highest extreme of severity (typical AD pattern) compared to the lowest extreme (minimal tau pattern).

On the discrete scale (Table 4), baseline tau-PET patterns were associated with greater longitudinal atrophy for typical AD and limbic predominant patterns but not the cortical predominant pattern in the entorhinal cortex. Baseline tau-PET pattern was associated with greater longitudinal atrophy for the typical AD pattern only in the neocortex. This result highlights a region-specific differential association between tau-PET patterns and atrophy. Typical AD and limbic predominant patterns showed increasing topographical correspondence between baseline tau-PET and atrophy over time while cortical predominant and minimal tau did not (visualized in Fig. 4). The two latter patterns showed marked atrophy in brain regions non-specific to the tau-PET patterns (e.g., entorhinal atrophy in cortical predominant; cortical atrophy in minimal tau), indicating that atrophy may not always regionally follow the different tau-PET patterns. Conversely, topographical correspondence has been reported between tau-PET and MRI in atrophy-based AD subtypes [46]. Combining findings from this study with ours may imply that heterogeneity of a downstream event (atrophy) may be reflected in an upstream event (tau pathology) but not vice versa. Downstream contributions of other neuropathologies towards atrophy may play a role in determining heterogeneity [47] and need to be considered as biomarkers for those pathologies become available. Altogether, considering tau pathology as a sole or main driver of neurodegeneration may be a simplification and understanding of disease heterogeneity requires a more unifying approach [48].

Across the continuous- and discrete-scale characterizations of tau-PET patterns, longitudinal atrophy associated with baseline tau pathology supports the hypothesis of tau pathology as a possible driver of atrophy [4, 7, 36, 49], observed across some but not necessarily all tau-PET patterns. Although findings from both the characterizations are consistent, the continuous-scale approach was significantly better than the discrete-scale one in being able to model longitudinal atrophy. While the continuous-scale approach characterizes the tau-PET patterns in terms of typicality and severity, two continuous dimensions of biological AD subtypes proposed by the recent conceptual framework [17], the conventional discrete-scale approach categorizes individuals into four discrete patterns based on the contribution of the entorhinal cortex and neocortex [26]. Typicality in the continuous-scale approach in fact factors in contributions from both the entorhinal cortex and neocortex used in the discrete-scale approach and further provides information on disease stage in terms of severity. The continuous-scale approach avoids arbitrary cutpoints, making it suitable for populations where the prevalence of different patterns is not well-known (e.g., beyond AD dementia including the AD continuum) and to small cohorts. The discrete-scale approach defines patterns based on a cutpoint (e.g., *Z*-score>1 relative to healthy Aβ− individuals in our study) [26, 35], influencing the prevalence of the identified patterns. Comparing across the four discrete-scale tau-PET patterns by the continuous-scale typicality and severity, we observed that each pattern was significantly different from the others in typicality as well as severity. It is thus, important to bear in mind that the discrete-scale tau-PET patterns representing heterogeneity are at different disease stages. Nevertheless, both approaches share some correspondence (Fig. 1): examining typicality, higher E:N may reflect a limbic predominant pattern while lower E:N may reflect a cortical predominant pattern; examining severity, higher global tau-PET SUVR may reflect a typical AD pattern while lower global tau-PET SUVR may reflect a minimal tau pattern. All previous subtyping methods in AD characterized heterogeneity on a discrete scale [14–16, 18], which is critical to delineate pattern-specific characteristics. However, discrete-scale characterizations often lack individual-level agreement [15]. A continuous-scale characterization of heterogeneity may be more useful as it is free from the assumption of pre-defined prevalence in a population. Hence, we encourage future studies to explore and validate new operationalizations of typicality and severity representing disease heterogeneity. Compared to the discrete-scale characterization of the tau-PET patterns which force-classifies each case into one of four categories (typical AD, limbic predominant, cortical predominant, minimal tau), the continuous-scale characterization additionally provides information on the extent of typicality and severity of each individual relative to others, thus, disentangling subtypes from disease stage, which could better inform the design of future clinical trials.

Furthermore, we noted differential profiles of the A/T/longitudinal-N biomarker scheme across tau-PET patterns (Fig. 5). Per definition, while the limbic predominant pattern demonstrated T+ in the entorhinal cortex and T− in the neocortex, the cortical predominant pattern demonstrated the opposite profile. This contrast may suggest a non-uniform sequence of tau accumulation across the tau-PET patterns. This aligns with the proposed hypothesis of alternative possible pathways for initiation/spread of tau pathology in the cortical predominant pattern [50]. All patterns showed some longitudinal neurodegeneration (adjusted for age), but only typical AD and limbic predominant patterns showed ≥50% prevalence of longitudinal N+. Combined with reports suggesting a preferential association of atrophy to tau pathology over Aβ [4, 51], this result may imply that atrophy may not entirely be tau-related and could be partly tau-independent, extending beyond the effect of normal aging. The minimal tau pattern presented a greater prevalence of T− both in the entorhinal and the neocortical regions. Relatively small proportion of the minimal tau cases show longitudinal N+. This may indicate the minimal tau group, while mostly reflecting Alzheimer’s pathologic change (A+/T−/longitudinal N−), could also contain cases with Alzheimer’s and concomitant suspected non-Alzheimer’s pathologic change (A+/T−/longitudinal N+). Whether the minimal tau pattern will remain as such or is a precedent manifestation of one of the other three tau-PET patterns will require analysis of longitudinal tau-PET. One caveat, however, is that the prevalence of A/T/longitudinal-N profiles varied widely depending on the cutpoint used (Supplementary Tables 2-3), an issue that is known in the field [1] which should be taken into account in future studies.

Our study has some limitations. Although the overall goal of our study was to understand the heterogeneity in tau-PET patterns across the AD continuum, cognitively normal individuals (Aβ+) were overrepresented. This dominance of the early stages of AD likely translated to the relatively less pronounced tau-PET patterns. Moreover, the ability of [^18^F] AV-1451 tracer in detecting tau pathology may be limited at these early disease stages [52]. Quantification of tau-PET patterns was based on tau-PET SUVR in the entorhinal and neocortex, regions with different availability of binding sites for this tracer [53]. Thus, alternative operationalizations of typicality, keeping in mind the relationship to severity, should be assessed in future work. Hippocampus, a key region in most neuropathological and MRI studies investigating heterogeneity in AD [12, 13, 25, 26, 54, 55], was not evaluated as its signal is confounded by off-target binding in tau-PET [27, 28, 56]. Thus, the limbic predominant pattern observed in our study may not directly be comparable to a limbic predominant subtype reported in postmortem investigation [12]. However, we have previously shown that tau-PET patterns based on the entorhinal cortex are similar to those based on hippocampus [15]. While we used cerebellum gray matter as a reference region for tau-PET, future studies would benefit from exploring alternative reference regions to minimize the spill-in effects [57]. Tau-PET patterns on the discrete scale may be influenced by the relatively lenient cutpoints used in our study. Given the large range of tau-PET cutpoints reported in the literature, future studies should focus on continuous characterization where possible or apply standardized thresholds [58]. Although we tracked the longitudinal atrophy changes relative to baseline tau pathology, we could not assess longitudinal tau-PET changes due to the limited samples of longitudinal tau-PET in the ADNI. Finally, considering the strict inclusion criteria in ADNI, the generalizability of our findings in a clinical setting or more heterogeneous population including non-amnestic clinical phenotypes remains to be validated.

In conclusion, we demonstrated that the associations are not the same between different tau-PET patterns and longitudinal atrophy in the AD continuum. Methodologically, we posit treating heterogeneity as a continuous phenomenon over the conventional discrete categorization. Together, our findings can have practical implications towards the design of clinical trials, development of targeted therapeutics, and ultimately, realization of precision medicine.

## Supplementary Information

**Additional file 1.**

## Data Availability

Source data used in this study are publicly available by the ADNI in the Laboratory of Neuro Imaging database upon registration. The data generated during the current study may be made available from the corresponding author on reasonable request.
