# Correction: Associations between different tau‑PET patterns and longitudinal atrophy in the Alzheimer’s disease continuum: biological and methodological perspectives from disease heterogeneity

**DOI:** 10.1186/s13195-023-01224-7

**Published:** 2023-04-13

**Authors:** Rosaleena Mohanty, Daniel Ferreira, Agneta Nordberg, Eric Westman, for the Alzheimer’s Disease Neuroimaging Initiative

**Affiliations:** 1 Division of Clinical Geriatrics, Center for Alzheimer Research, Department of Neurobiology, Care Sciences and Society, Karolinska Institutet, Blickagången 16, 14152 Huddinge, Sweden; 2 Department of Radiology, Mayo Clinic, Rochester, MN USA; 3 Theme Aging, Karolinska University Hospital, Stockholm, Sweden; 4 Department of Neuroimaging, Centre for Neuroimaging Sciences, Institute of Psychiatry, Psychology and Neuroscience, King’s College London, London, UK

**Correction** **:** **Alz Res Therapy 15, 37 (2023)**

10.1186/s13195-023-01173-1

Following publication of the original article [1], the authors identified an error in Fig. 3 (subfigure on the right has missing labels). The labels match what is reported in the text (Results section). The correct figure is given below.

**Fig. 3:**
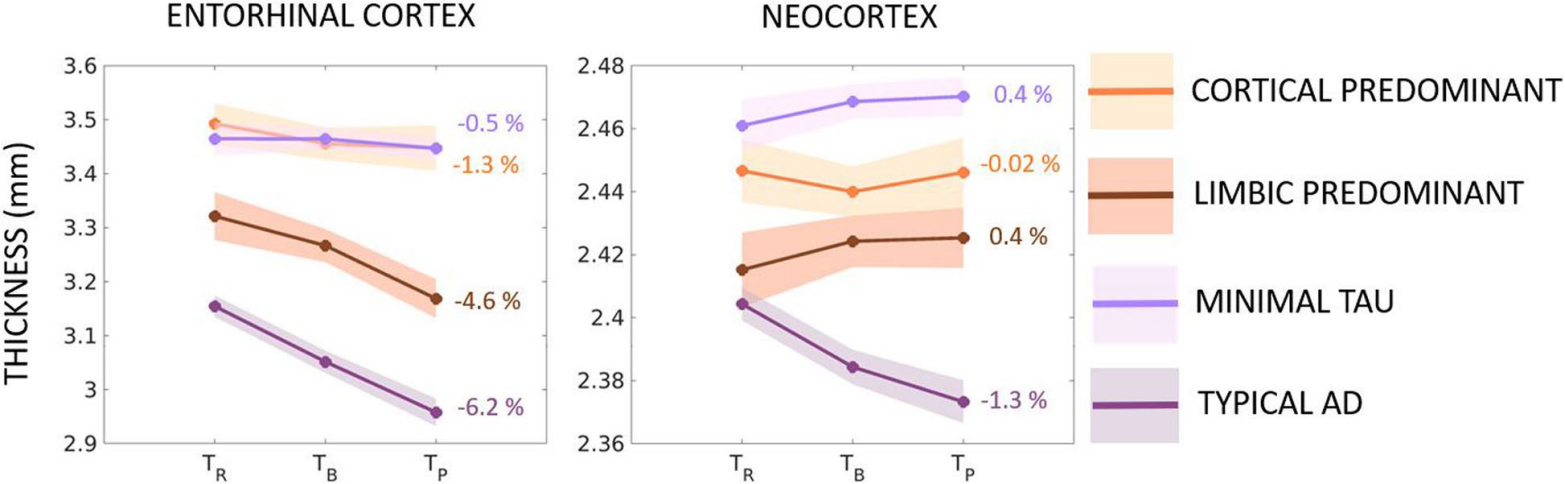
Longitudinal changes in atrophy relative to baseline tau-PET patterns (discrete scale) in the AD continuum. Estimated longitudinal atrophy (thickness) estimated by linear mixed effects model for the entorhinal cortex and neocortex stratified by levels of tau-PET patterns on the discrete scale including typical AD, limbic predominant, cortical predominant, and minimal tau patterns. Shaded regions represent the 95% confidence interval; percentages indicate the overall change in thickness per group over the period between retrospective and prospective timepoints

The original article [1] has been updated.
