# Supplementary material for: Associations between different tau-PET patterns and longitudinal atrophy in the Alzheimer’s disease continuum: biological and methodological perspectives from disease heterogeneity
Source: Alzheimers Res Ther. 2023 Feb 22;15:37. doi: 10.1186/s13195-023-01173-1 (PMC9945609; doi:10.1186/s13195-023-01173-1)
Supplement: Supplementary file 1 — Additional file 1. [file 13195_2023_1173_MOESM1_ESM.docx]

***Associations between different tau-PET patterns and longitudinal atrophy in the Alzheimer’s disease continuum***

***Biological and methodological perspectives from disease heterogeneity***

**Supplementary Material**

**Supplementary Figure 1.** Study design investigating biological and methodological perspectives of tau-PET patterns


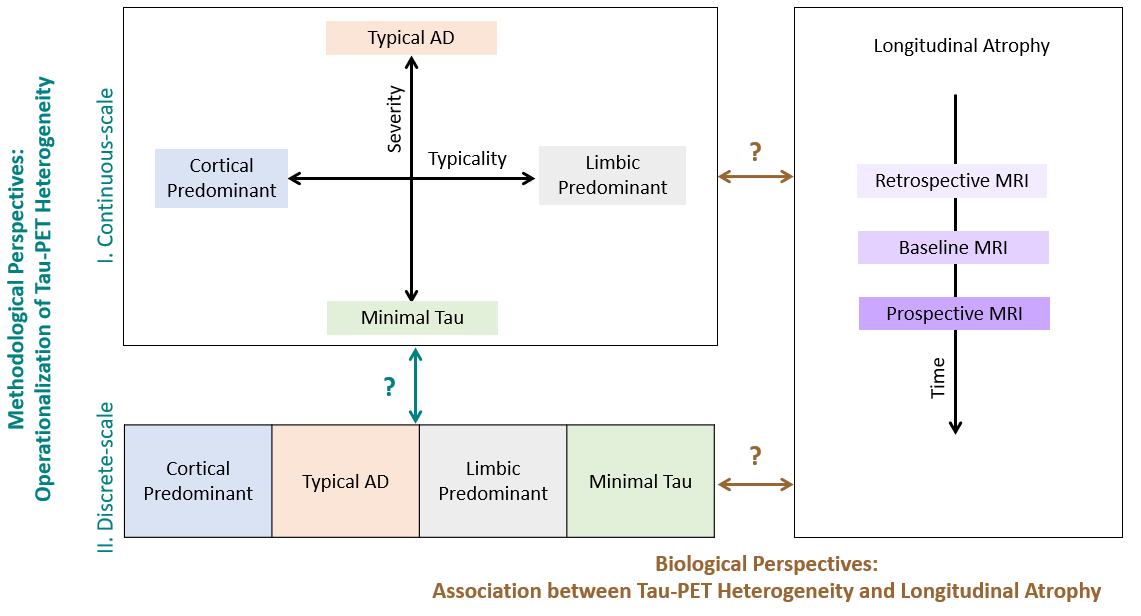


**Supplementary Figure 2.** Regions of interest for tau pathology and brain atrophy


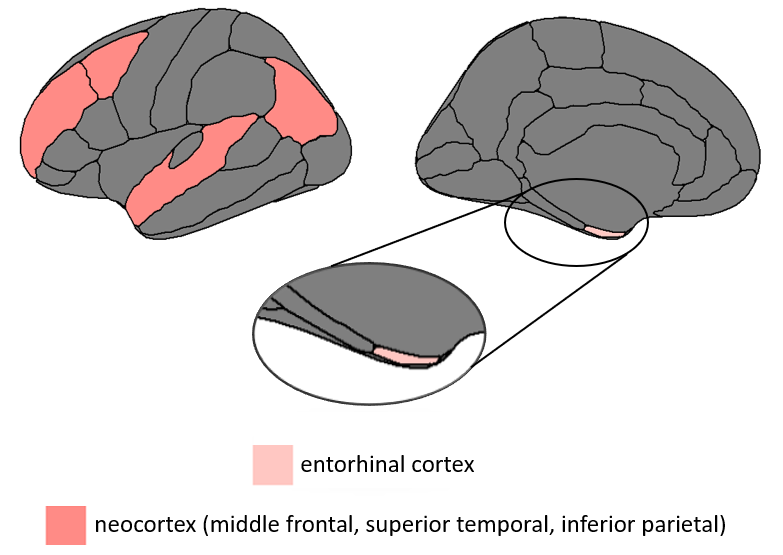


**Supplementary Figure 3.** Distribution of entorhinal and neocortical tau-PET SUVR across the AD continuum compared to healthy controls


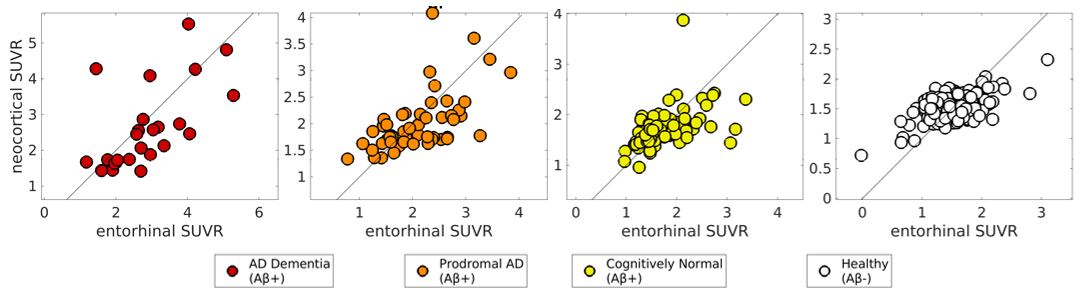


In healthy individuals, the entorhinal and neocortical SUVR are comparable (evident from tau-PET SUVR along the reference proportional diagonal line). In comparison, along the AD continuum, there is an imbalance of tau-PET SUVR between the two regions (i.e., heterogeneity in tau pathology) which increases from the cognitively normal to prodromal AD to AD dementia stag (evident from deviation of tau-PET SUVR from the reference proportional diagonal line). This imbalance extends in both directions in the form of relatively higher entorhinal SUVR compared to neocortical SUVR in some cases and relatively lower entorhinal SUVR compared to neocortical SUVR in other cases.

**Supplementary Section 1. Subtyping algorithm to characterize tau-PET patterns on a discrete scale** (8)

*Measure:* cortical tau-PET SUVR

*Regions:* medial temporal lobe represented by entorhinal cortex; neocortex represented by middle frontal, superior temporal, inferior parietal regions; each region averaged across both cerebral hemispheres (**Supplementary Figure 2**).

*Method:*

(i) Z-score normalization: All regional tau-PET SUVR values were converted into Z-scores by comparing against the healthy (Aβ-) reference group.

(ii) Abnormality Identification: Z-scores of tau-PET SUVR in the entorhinal cortex (Z_E_), and neocortex (average of frontal, temporal_,_ parietal regions, Z_N_) of individuals were calculated based on the normative data. A cutpoint of Z-score>1.0 determined the presence of abnormal tau pathology in each ROI. This corresponded to cutpoints of 1.87 and 1.52 for the entorhinal cortex and neocortex respectively.

(iii) Classification: Four discrete patterns were identified. If Z_E_>1 and Z_N_>1, then individuals were classified as the *typical AD* pattern. If Z_E_>1 and Z_N_≤1, then individuals were classified as the *limbic predominant* pattern. If Z_E_≤1 and Z_N_>1, then individuals were classified as *cortical predominant* pattern. If Z_E_≤1 and Z_N_≤1, then individuals were classified as the *minimal tau* pattern.

**Supplementary Section 2. Tau positivity (T+) in tau-PET patterns**

Regional tau positivity was evaluated and compared two ways:

(i) *Byun’s cutpoint:* tau positivity was defined as the Z-score of tau-PET SUVR > 1 relative to the healthy (Aβ-) reference group, i.e., Z_E_>1 for the entorhinal cortex and Z_N_>1 for the neocortex (middle frontal, superior temporal, inferior parietal) based on a previous subtyping study (8). The corresponding cutpoints were 1.87 and 1.52 for the entorhinal cortex and neocortex respectively.

(iii) *Braak staging-based cutpoint:* tau positivity was defined for the entorhinal (Braak stage I) as PVC tau SUVR > 1.129 (the hippocampus corresponding to Braak stage II was excluded due to potential off-target binding in the region) and for cortical brain areas including frontal cortex, parietal cortex, occipital cortex, transverse, superior temporal cortex, precuneus, banks of superior temporal sulcus, precentral gyrus, postcentral gyrus, paracentral gyrus, cuneus, pericalcarine (Braak stages V-VI) as PVC tau SUVR > 1.873 based on a previous tau-PET study (9).

Consistent with our regions of interest (entorhinal cortex and neocortex, **Supplementary Figure 2**), our main findings correspond to the results from Byun’s cutpoints above.

**Supplementary Section 3. Longitudinal neurodegeneration (N_R_+, N_B_+, N_P_+) positivity in tau-PET patterns**

Regional longitudinal neurodegeneration positivity was evaluated and compared two ways:

(i) *Byun’s cutpoint:* neurodegeneration positivity was defined as the Z-score of MRI-based thickness < 1 relative to the healthy (Aβ-) reference group, i.e., Z_E_<1 for the entorhinal cortex and Z_N_<1 for the neocortex (middle frontal, superior temporal, inferior parietal) based on a previous subtyping study (8). This procedure was repeated at longitudinally (retrospective, baseline, prospective timepoints).

(ii) *Meta ROI-based cutpoint:* neurodegeneration positivity was defined in a meta ROI in the medial temporal lobe (entorhinal, inferior temporal, middle temporal, fusiform) as thickness < 2.67 mm based on a previous study establishing imaging-based cutpoints in AD (10). Due to unavailability of a specific threshold for the cortex, the same threshold of 2.67 mm was applied to the neocortex (middle frontal, superior temporal, inferior parietal). This procedure was repeated at longitudinally (retrospective, baseline, prospective timepoints).

Consistent with our regions of interest (entorhinal cortex and neocortex, **Supplementary Figure 2**), our main findings correspond to the results from Byun’s cutpoints above.

**Supplementary Section 4. Longitudinal cognition in relation to baseline tau pathology and longitudinal neurodegeneration in tau-PET patterns**

*Methods:* In 61 individuals followed up across all three timepoints, we examined the association between change in cognition (dependent variable) and tau pathology at baseline, change in atrophy or thickness and interval between timepoints (independent variables) using multiple regression models. The regions of interest were consistent with those of the main study and included the entorhinal cortex and the neocortex (**Supplementary Figure 2**).

**Supplementary Table 1.**  Association of longitudinal cognitive changes with baseline tau pathology and longitudinal atrophy across the tau-PET patterns (discrete-scale)

| **Cognitive outcome and timepoints for change in cognition and MRI** | | **Entorhinal Cortex** | | | | **Neocortex** | | | |
| --- | --- | --- | --- | --- | --- | --- | --- | --- | --- |
|  |  | **TAD**  **(N=25)** | **LP**  **(N=5)** | **CP**  **(N=13)** | **MT**  **(N=18)** | **TAD**  **(N=25)** | **LP**  **(N=5)** | **CP**  **(N=13)** | **MT**  **(N=18)** |
| MMSE | Retrospective to Baseline change | 0.23 | 0.94 | 0.44 | 0.08 | 0.15 | 0.92 | 0.15 | 0.17 |
|  | Baseline to Prospective  change | 0.45 *****τ | 0.82 | 0.31 | 0.29 | 0.48 *****τα | 0.81 | 0.35 | 0.19 |
| ADNI-MEM | Retrospective to Baseline change | 0.14 | 0.99 | 0.28 | 0.53 *****τ | 0.32  *****τ | 0.97 | 0.21 | 0.51 ***** |
|  | Baseline to Prospective  ADNI-MEM change | 0.39 ***** | 0.68 | 0.34 | 0.21 | 0.34 ***** | 0.66 | 0.19 | 0.47  *****τ |
| ADNI-EF | Retrospective to Baseline change | 0.79  ***** | - | 0.4 | 0.32 | 0.78 ***** | - | 0.68 *****α | 0.25 |
|  | Baseline to Prospective  ADNI-EF change | 0.15 | - | 0.29 | 0.21 | 0.03 | - | 0.51 | 0.39 |

Significant associations are expressed in terms of R^2^, the coefficient of determination and values corresponding to *p*≤0.05 are marked by *; Associations with significant contributions from baseline tau only (τ), longitudinal atrophy/thickness change (α) and baseline tau along with longitudinal atrophy/thickness change (τα) are marked. Change in atrophy/thickness from timepoint 1 to timepoint 2 $=\left( \begin{matrix} \frac{thickness at timepoint 1 - thickness at timepoint 2}{thickness at timepoint 1} \end{matrix} \right)$; All associations are accounted for interval between timepoints. AD = Alzheimer’s disease; Analyses for missing data were omitted due to lack of sample size and marked as blank (-); TAD = typical AD pattern; LP = limbic predominant pattern; CP = cortical predominant pattern; MT = minimal tau pattern; MMSE=mini mental state examination; ADNI-MEM=composite cognitive scores for memory; ADNI-EF= composite cognitive scores for executive function.

*Results:* We observed a differential association across tau-PET patterns. Typical AD most commonly showed significant effects across the entorhinal and neocortex. For global cognition, both baseline tau and prospective atrophy changes contributed to prospective MMSE changes. For the memory composite score (ADNI-MEM), baseline tau pathology primarily was the driver of both retrospective and prospective cognitive changes. For the executive function composite score (ADNI-EF), retrospective atrophy changes contributed towards retrospective cognitive changes. Associations corresponding to limbic-predominant pattern should be cautiously interpreted due to limited sample size.

**Supplementary Figure 4.** Contribution of different cortical regions towards the cortical predominant tau-PET pattern


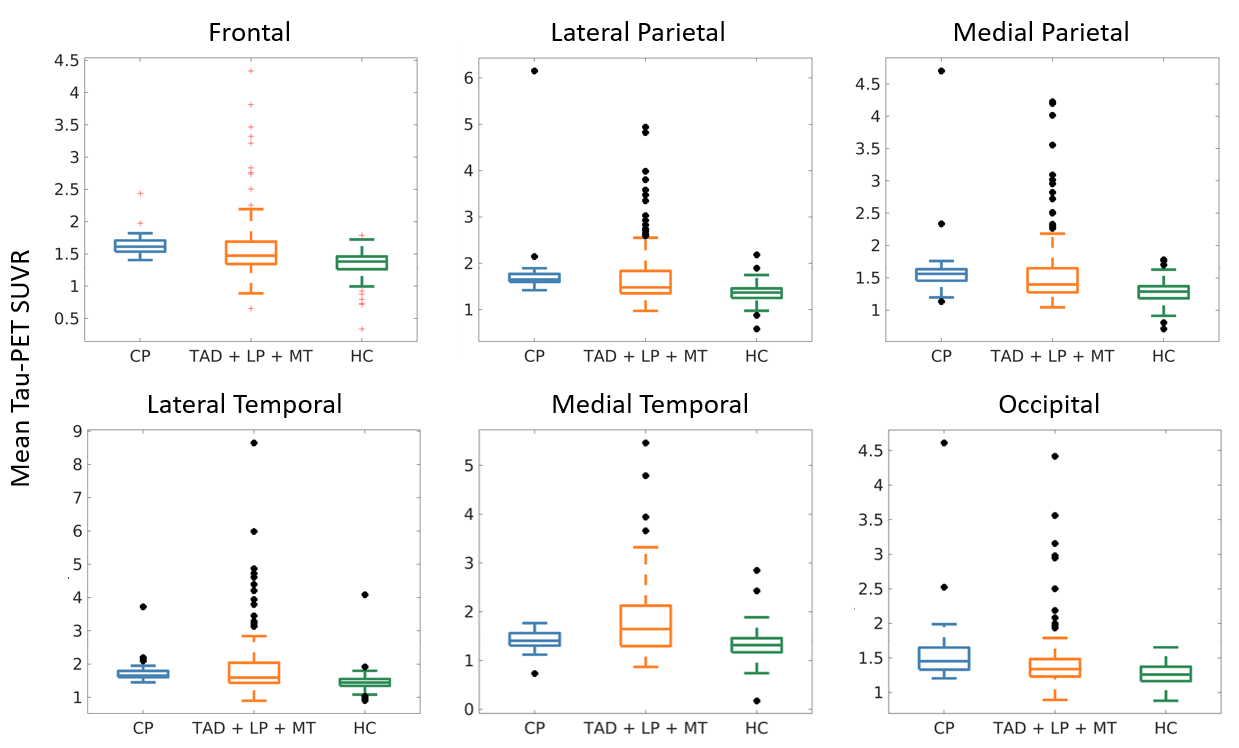


For all regions except the medial temporal, the CP and non-CP (TAD + LP + MT) patterns showed significantly higher tau-PET SUVR than in HC (*p* < 0.0001) based on independent two-sample t-tests. And the tau-PET SUVR of CP did not significantly differ from that of non-CP in any of the regions (*p* > 0.05). For the medial temporal region only, the tau-PET SUVR for CP did not differ significantly from the HC (*p* = 0.1) but differed significantly from the non-CP (*p* = 0.0021). These findings suggest that CP is characteristically different from the other patterns primarily in the medial temporal tau pathology. CP=cortical predominant, TAD=typical Alzheimer’s; LP=limbic predominant; MT=minimal tau; HC=healthy control.

**Supplementary Figure 5.** Continuous-scale tau-PET patterns (typicality and severity) stratified by the four discrete-scale tau-PET patterns


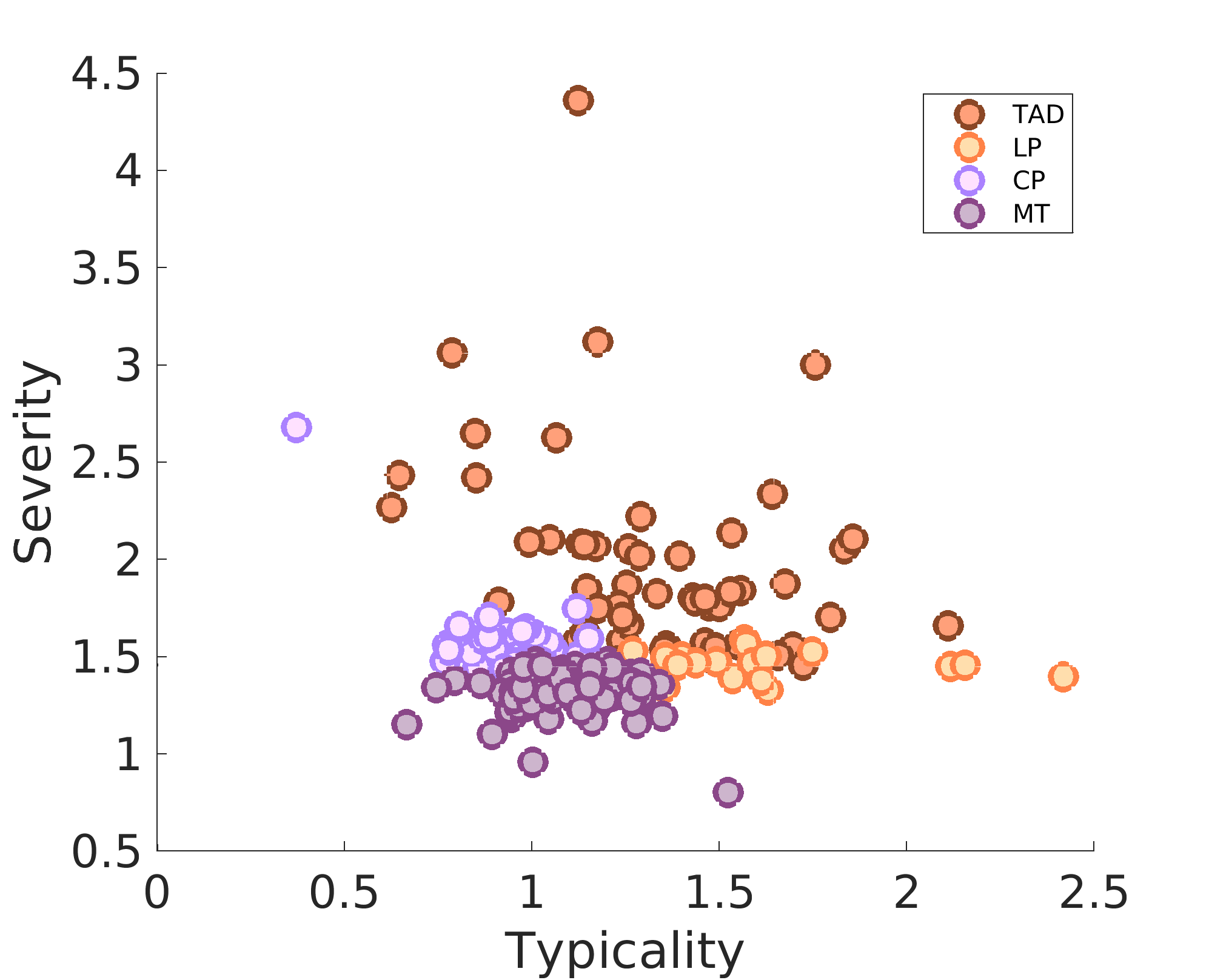


CP=cortical predominant, TAD=typical Alzheimer’s; LP=limbic predominant; MT=minimal tau; HC=healthy control.

**Supplementary Table 2.** Comparison of cutpoints to determine baseline tau positivity (T+) across tau-PET patterns

| **All Aβ+ subjects at baseline (N=173)** | | | | | |
| --- | --- | --- | --- | --- | --- |
| **Region** | **Method** | **TAD (N=57)** | **LP (N=21)** | **CP (N=31)** | **MT (N=64)** |
| **Medial temporal**  **(T+ %)** | Byun | 100 | 100 | 0 | 0 |
|  | Braak | 100 | 100 | 100 | 93.7 |
| **Cortex (T+ %)** | Byun | 100 | 0 | 100 | 0 |
|  | Braak | 85.9 | 14.3 | 83.9 | 3.12 |

Cutpoints corresponding to the two methods compared are described in Supplementary Section 2. T+=tau positivity; AD=Alzheimer’s disease; Aβ=amyloid-beta; TAD=typical AD pattern; LP=limbic predominant pattern; CP=cortical predominant pattern; MT=minimal tau pattern. Consistent with our regions of interest (entorhinal cortex and neocortex, Supplementary Figure 1), findings in the main text correspond to the results from Byun’s cutpoints above.

**Supplementary Table 3.** Comparison of cutpoints to determine longitudinal neurodegeneration positivity in tau-PET patterns

| **Subcohort (Aβ+) with longitudinal MRI at all three timepoints (N=61)** | | | | | | | | | | | | | |
| --- | --- | --- | --- | --- | --- | --- | --- | --- | --- | --- | --- | --- | --- |
|  | | **TAD (N=38)** | | | **LP (N=8)** | | | **CP (N=19)** | | | **MT (N=29)** | | |
| **Region** | **Method** | **T_R_** | **T_B_** | **T_P_** | **T_R_** | **T_B_** | **T_P_** | **T_R_** | **T_B_** | **T_P_** | **T_R_** | **T_B_** | **T_P_** |
| **Medial temporal (N+ %)** | Byun | 56 | 84 | 84 | 60 | 60 | 60 | 15.4 | 30.8 | 15.4 | 27.8 | 38.9 | 22.2 |
|  | Meta ROI | 40 | 68 | 68 | 20 | 60 | 60 | 15.4 | 30.8 | 23.1 | 11.1 | 16.7 | 11.1 |
| **Cortex (N+ %)** | Byun | 48 | 68 | 64 | 40 | 60 | 60 | 38.5 | 46.1 | 38.5 | 22.2 | 38.9 | 27.8 |
|  | Meta ROI | 80 | 96 | 96 | 100 | 100 | 100 | 76.9 | 84.6 | 84.6 | 77.8 | 88.9 | 88.9 |

Cutpoints corresponding to the two methods compared are described in Supplementary Section 3. N+=neurodegeneration positivity; N+ values are adjusted for age at each timepoint; AD=Alzheimer’s disease; Aβ=amyloid-beta; TAD=typical AD pattern; LP=limbic predominant pattern; CP=cortical predominant pattern; MT=minimal tau pattern; T_R_=retrospective timepoint; T_B_=baseline timepoint; T_P_=prospective timepoint; Consistent with our regions of interest (entorhinal cortex and neocortex, Supplementary Figure 1), findings in the main text correspond to the results from Byun’s cutpoints above.

**Supplementary Table 4.** A/T/longitudinal-N biomarker scheme corresponding to the tau-PET patterns in the AD continuum

| **AD continuum (100% A+) at baseline (N=173)** | | | | | | |
| --- | --- | --- | --- | --- | --- | --- |
|  | | | **TAD (N=57)** | **LP (N=21)** | **CP (N=31)** | **MT (N=64)** |
| **Entorhinal Cortex** | **T+ %** | | 100 | 100 | 0 | 0 |
|  | **N+ %** | | 63.2 | 52.4 | 29.0 | 28.1 |
| **Neocortex** | **T+ %** | | 100 | 0 | 100 | 0 |
|  | **N+ %** | | 50.9 | 42.9 | 41.9 | 26.6 |
| **Subcohort of AD continuum (100% A+) with longitudinal MRI at all three timepoints (N=61)** | | | | | | |
|  | | | **TAD (N=38)** | **LP (N=8)** | **CP (N=19)** | **MT (N=29)** |
| **N+ %**  **Entorhinal Cortex** | | **T_R_** | 56 | 60 | 15.4 | 27.8 |
|  |  | **T_B_** | 84 | 60 | 30.8 | 38.9 |
|  |  | **T_P_** | 84 | 60 | 15.4 | 22.2 |
| **N+ %**  **Neocortex** | | **T_R_** | 48 | 40 | 38.5 | 22.2 |
|  |  | **T_B_** | 68 | 60 | 46.1 | 38.9 |
|  |  | **T_P_** | 64 | 60 | 38.5 | 27.8 |

A+=Aβ positivity with global PET Aβ SUVR; T+=tau positivity with Byun’s cutpoint; N+=neurodegeneration positivity with Byun’s cutpoint; N+ values are adjusted for age at each timepoint; AD=Alzheimer’s disease; TAD=typical AD pattern; LP=limbic predominant pattern; CP=cortical predominant pattern; MT=minimal tau pattern.

**References**

1. Joshi AD, Pontecorvo MJ, Clark CM, Carpenter AP, Jennings DL, Sadowsky CH, et al. Performance characteristics of amyloid PET with florbetapir F 18 in patients with Alzheimer’s disease and cognitively normal subjects. J Nucl Med. 2012;53(3):378–84.

2. Muehlboeck J, Westman E, Simmons A. TheHiveDB image data management and analysis framework. Front Neuroinform. 2014;7:49.

3. Reuter M, Schmansky NJ, Rosas HD, Fischl B. Within-subject template estimation for unbiased longitudinal image analysis. Neuroimage. 2012;61(4):1402–18.

4. Desikan RS, Ségonne F, Fischl B, Quinn BT, Dickerson BC, Blacker D, et al. An automated labeling system for subdividing the human cerebral cortex on MRI scans into gyral based regions of interest. Neuroimage. 2006;31(3):968–80.

5. Greve DN, Salat DH, Bowen SL, Izquierdo-Garcia D, Schultz AP, Catana C, et al. Different partial volume correction methods lead to different conclusions: an 18F-FDG-PET study of aging. Neuroimage. 2016;132:334–43.

6. ROUSSET, OG. Correction for partial volume effects in PET : principle and validation. J Nucl Med [Internet]. 1998 [cited 2020 Feb 18];39:904–11. Available from: http://ci.nii.ac.jp/naid/10025136344/en/

7. Mohanty R, Mårtensson G, Poulakis K, Muehlboeck J-S, Rodriguez-Vieitez E, Chiotis K, et al. Comparison of subtyping methods for neuroimaging studies in Alzheimer’s disease: a call for harmonization. Brain Commun. 2020;2(2):fcaa192.

8. Byun MS, Kim SE, Park J, Yi D, Choe YM, Sohn BK, et al. Heterogeneity of regional brain atrophy patterns associated with distinct progression rates in Alzheimer’s disease. PLoS One. 2015;10(11):e0142756.

9. Maass A, Landau S, Baker SL, Horng A, Lockhart SN, La Joie R, et al. Comparison of multiple tau-PET measures as biomarkers in aging and Alzheimer’s disease. Neuroimage. 2017;157:448–63.

10. Jack Jr CR, Wiste HJ, Weigand SD, Therneau TM, Lowe VJ, Knopman DS, et al. Defining imaging biomarker cut points for brain aging and Alzheimer’s disease. Alzheimer’s Dement. 2017;13(3):205–16.
